# Histopathological Study of Esophageal Infection with *Gasterophilus pecorum* (*Diptera: Oestridae*) in Persian Onager (*Equus hemionus onager*)

**Published:** 2017-09-08

**Authors:** Seyed Mohammad Hoseini, Bahram Ali Zaheri, Mohamad Ali Adibi, Hooman Ronaghi, Amir Hossein Moshrefi

**Affiliations:** 1 Department of Pathobiology, Babol Branch, Islamic Azad University, Babol, Iran; 2 Department of Environmental Protection Semnan, Semnan, Iran; 3 Department of Microbiology, Faculty of Specialized Veterinary Science, Science and Research Branch, Islamic Azad University, Tehran, Iran; 4 Young Researchers and Elite Club, Babol Branch, Islamic Azad University, Babol, Iran

**Keywords:** Persian onager, *Gasterophilus pecorum*, *Epithelial destruction*

## Abstract

**Background:**

The larval stages of *Gasterophilus* are obligate parasites in the gastrointestinal tract of equine accountable for pathologic ulcers in the Persian onager gastrointestinal. The aim of the current report was to study the histopathological change with *G. pecorum* larvae in the esophagus of a Persian onager.

**Methods:**

This study was performed in Iranian Zebra propagation and breeding site in Khartouran National Park, southeast of Shahrud City, Semnan Province, Iran in 2014. Following a necropsy with specific refer to esophagus of one adult female Persian onager were transmitted to the laboratory. After autopsy, parasites collected from the esophagus were transmitted into 70% alcohol. For histopathological investigation, tissue samples were collected from the esophagus. The tissues were fixed in 10% buffered formalin, and conformity routine processing, there were stained with Hematoxylin and eosin.

**Results:**

After clarity by lactophenol parasites were identified as *G. pecorum*. Microscopic recognition contained hyperemia, inflammatory cell infiltration, epithelial destruction, esophageal gland hyperplasia.

**Conclusion:**

This is the first survey of *G. pecorum* and histopathological study in the Persian onager esophagus in the world.

## Introduction

The Persian onager (*Equus hemionus onager*), a wild donkey endemic to Iran, is classified as critically endangered on the International Union for Conservation of Nature Red List. The Asian wild donkeys were confined in successive periods but ecology of the two residual crowds, determined in preserved region in Touran National Park and Bahram-e-Goor Reserve (1).

The genus *Gasterophilus* (Diptera: Oestridae) contains nine species. Equids are hosts to the larvae of the *Gasterophilus* type causing gastrointestinal myiasis. *Gasterophilus* is specified by dysphasia, gastrointestinal ulcer ations, intestinal obstruction or volvulus, rectal prolapses, anemia, diarrhea and digestive disturbances.

The adult flies are not parasitic and are large, 11–15mm in length. Adult *Gasterophilus* spp. flies lay their eggs to host hairs. *G. pecorum* is an exception as females lay their eggs in brown-haired person, leaves, and stalks of plants (2, 3).

After hatching, the larvae tunnels into the tissue of the host, larvae at the first stage attain the oral cavity of equine passively (*G. intestinalis*, *G. pecorum*) or actively, the first stage larvae hatch and moult to L2, which can be available in various regions of the gastrointestinal tract, and in L3 remains dependent to the mucosa for 8–10 months (4, 5).

*Gasterophilus pecorum*, *G. inermis*, and *G. haemorrhoidalis* are just reported in finite regions of Europe and Eastern Countries (6).

The damage the bot fly reasons happens after the larvae arrive the animal’s mouth and gastrointestinal tract. When the first instar larvae tunnel into the mouth, the horse may experience intense inflammation, as well as the expansion of pus pockets and loosened teeth. Loss of appetite may develop due to the larva’s resident. As the second and third instar larvae reside the gastrointestinal tract and bind to the stomach and intestine, variable complications can occur. Severe infestation of these larvae can cause anemia, esophageal paralysis, ulcerated stomach, chronic gastritis, stomach rupture and squamous cell tumors (7).

Esophageal disorders, exception obstruction is not common to observe in equine. Little is known about the parasite spectrum of this species. Accordingly, there was severity of the infection in this area of robot flies.

The aim of the current report was to study the histopathological change with *G. pecorum* larvae in the esophagus of a Persian onager.

## Materials and Methods

This study was performed in Iranian Zebra propagation and breeding site in Khartouran National Park, southeast of Shahrud City, Semnan Province, Iran (latitude 36.736536, longitude 55.700684) in 2014, with a temperate climate. A cervical vertebral fracture after collision with a fence was diagnosed as cause of death of the 20-yr-old female Persian onager. Probably it escaped from something and did not see a fence and subsequently broken neck lead to death.

Following a field, necropsy gastrointestinal system was attentively removed and transferred directly to the Laboratory of Veterinary Diagnostic Medicine of the Islamic Azad University-Babol Branch for histopathologic and parasitological examination.

The esophagus was assayed for parasite infections. The large changes were recorded, and myiasis was collected and transferred into 70% alcohol (Jahan Alcohol Teb Co., Arak 454546, Iran). The parasites were detected as *G. pecorum* by light microscope with referral to key Zumpt keys (8) using stereo microscopes with 10X to 40X magnification. Tissue samples and myiasis were used for histopathological examinations fix the tissue immediately in 10% buffered formalin, paraffin-embedded and sections were cut using a rotary microtome (Leitz, 1512, Germany) at 5μm and stained with hematoxylin and eosin (H and E).

The study was approved by the Animal Ethics Committee of Islamic Azad University, Babol Branch, Babol, Iran.

## Results

**Fig. 1.**
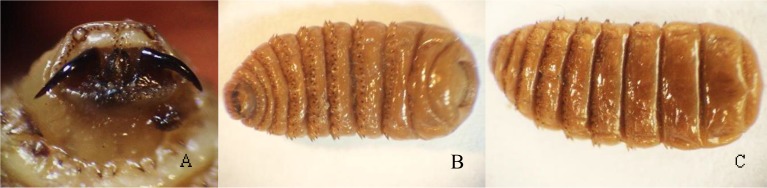
Third larval stage of *Gasrophilus pecorum* A. Ventral view of pseudocephalon B. ventral view C. dorsal view

Esophageal necropsy revealed that the onager was infected to myiasis. Totally, 87 third larval stage of *G. pecorum* were removed from esophageal tissue of the animal (Fig. 1).

**Fig. 2.**
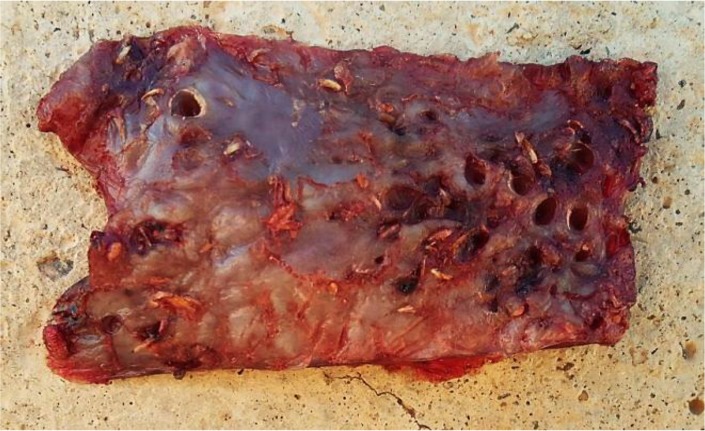
*Gasterphilus* larvae on the esophageal mucosal membrane of an onager

The third larval stage of *G. pecorum* was the arrangement of denticles on the pseudocephalon into 3 groups, 2 lying laterally and a third centrally in front of the mouth hooks (Fig. 2).

**Fig. 3.**
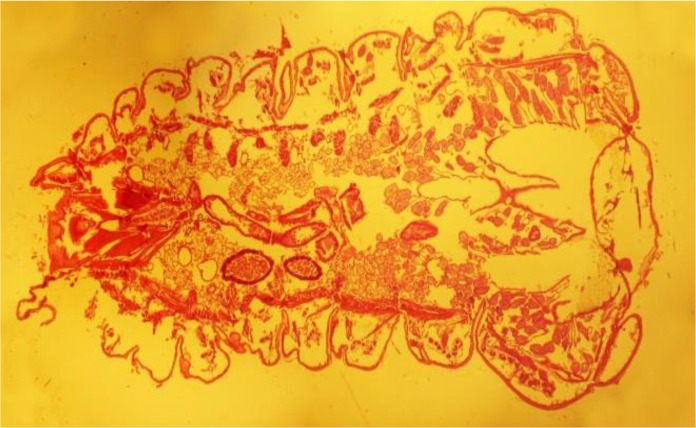
Cross-sections *Gasterphilus* larvae (10×), H and E

**Fig. 4.**
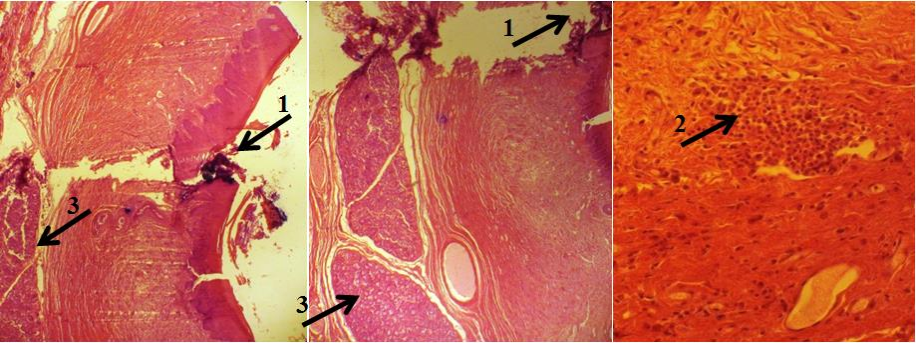
Cross sections of intraluminal myiasis, Inflammatory cell infiltration (1), epithelial destruction (2), Esophageal gland hyperplasia (3). H and E. ×40

We found gastric myiasis caused by *G. pecorum* (365 larvae). The histopathological study revealed different part of myiasis (Fig. 3). Microscopic examination showed epithelial destruction, esophageal gland hyperplasia, hyperemia, lymphocyte and macrophage infiltration in mucosa and submucosa of esophagus of the *Equus hemionus* infected by *Gasterophilus* (Fig. 4).

## Discussion

Botfly infestation has been reported in different countries can cause economic losses in domestic animals. The presence of *Gasterophilus* species has been much studied in Asia extensively as the most pathogenic *Gasterophilus* species on horses.

Accomplished investigations in different parts of the world, incidence ranged from 11% to 100% including 11.1% in Israel (9), 12.3% in Sweden (10), 43% in Ireland (11), 34% in France (12), 53% in England and Wales (13), 58% in Belgium (14), 82.2% in Italy (6), 65% in Switzerland (15), 98.7% in Kentucky, USA (16) and 100% in Morocco (17, 18).

Bots in the alimentary tract were identified as third stage larvae associated with ulcers include *G. pecorum*, *G. nigricornis* and *G. nasalis* (19). *Gasterophilus pecorum* has been studied in Asia where it is regarded as the most pathogenic *Gasterophilus* species on horses (8).

A study in China was done on the diagnosis of the larval *Gasterophilus* species in 90 equines, from 2008 to 2013 revealed the all-90 (100%) equines were infested via larval *Gasterophilus*, and 3723 secondary instar larvae (L2) as well as 63778 third instar larvae (L3). Six types of *Gasterophilus* were recognized include *G. pecorum* 88.94%, *G. nigricornis* 4.94%, *G. nasalis* 3.93%, *G. haemorrhoidalis* 1.91%, *G. intestinalis* 0.19%, and *G. inermis* 0.087% (20). In Iran, the onager was infected by myiasis (*G. pecorum*) and nematode (*Habronema muscae*) (21).

According to our results, *G. pecorum* is more adaptable to the local environment in Khartouran National Park. The association with this unique comportment and the desert steppe ecosystem can help describe the situation.

Water availability limits the activity area of wild animals in a region such as Khartouran National Park, which has high evaporation, limited surface runoff, and low precipitation. A study in Kalamaili showed that the oviposition sites of *G. pecorum* were often near a water source (3). Frequent drinking at water sources may increase the risk of *G. pecorum* infection. Thus, the equids in arid desert grasslands have a higher intensity of *Gasterophilus* spp.

## Conclusion

This is the first report of this parasite and histopathological study in the Persian onager esophagus in the world.

## References

[1] Tatin L Darreh-Shoori BF Tourenq C Tatin D Azmayesh B (2003) The last populations of the Critically Endangered onager *Equus hemionus* onager in Iran: urgent requirements for protection and study. Oryx. 37( 4): 488– 491.

[2] Gökçen A Sevgili M Altaş MG Camkerten İ (2008) Presence of *Gasterophilus* species in Arabian horses in Sanliurfa region. Turkiye Parazitol Derg. 32: 337– 339. 19156607

[3] Liu SH Hu DF Li K (2015) Oviposition site selection by *Gasterophilus pecorum* (Diptera: Gasterophilidae) in its habitat in Kalamaili Nature Reserve, Xinjiang, China. Parasite. 22: 34. 26621549 10.1051/parasite/2015034 PMC4664853

[4] Studzińska MB Wojcieszak K (2009) *Gasterophilus* sp. botfly larvae in horses from the south-eastern part of Poland. Bull Vet Inst Pulawy. 53: 651– 655.

[5] Ganjali M Keighobadi M (2016) A Rare Case of Gastric Myiasis in a Lion Caused by *Gasterophilus intestinalis* (Diptera: Gasterophilidae)-Case Report. J Arthropod Borne Dis. 10( 3): 421– 3. 27308300 PMC4906748

[6] Otranto D Milillo P Capelli G Colwell DD (2005) Species composition of *Gasterophilus* spp. (Diptera, Oestridae) causing equine gastric myiasis in southern Italy: parasite biodiversity and risks for extinction. Vet Parasitol. 133( 1): 111– 118. 15978726 10.1016/j.vetpar.2005.05.015

[7] Mashayekhi M Ashtari B (2013) Study of *Gasterophillus* role in equine gastric ulcer syndrome in Tabriz area. Bull Env Pharmacol Life Sci. 2( 12): 169– 172.

[8] Zumpt F (1965) Myiasis in man and animals in the Old World. A textbook for physicians, veterinarians and zoologists, Butterworths, London.

[9] Sharir B Pipano E Markovics A Danieli Y (1987) Field studies on gastrointestinal infestation in Israeli Horses. Isr J Vet Med. 43: 223– 227.

[10] Hoglund J Ljungstrom BL Nilsson O Lundguist H Osterman E Uggla A (1997) Occurrence of Gasterophilus intestinalis and some parasitic nematodes of horses in Sweden. Acta Vet Scand. 38: 157– 165. 9257451 10.1186/BF03548495 PMC8057032

[11] Sweeney HJ (1990) The prevalence and pathogenicity of Gasterophilus intestinalis larvae in horses in Ireland. Irish Vet J. 43: 67– 73.

[12] Bernard N Collobert C Tariel G Lamidey C (1994) Epidemiological survey of bot infection in horses at necropsy in Normandy from April 1990 to March 1992. Rec Med Vet. 170: 231– 235.

[13] Edwards GT (1982) The prevalence of Gasterophilus intestinalis in horses in northern England and Wales. Vet Parasitol, 11: 215– 222. 6891853 10.1016/0304-4017(82)90044-9

[14] Agneessens J Engelen S Debever P Vercruysse J (1998) Gasterophilus intestinalis infections in horses in Belgium. Vet Parasitol. 77: 199– 204. 9746291 10.1016/s0304-4017(98)00106-x

[15] Brocard P Pfister K (1991) The epidemiology of gasterophilosis of horses in Switzerland. Schweiz Arch Tierheilkd. 133: 409– 416. 1771404

[16] Drudge JH Lyons ET Wyant ZN Tolliver SC (1975) Occurrence of second and third instars of Gasterophilus intestinalis and Gasterophilus nasalis in stomachs of horses in Kentucky. Am J Vet Res. 36: 1585– 1588. 1190600

[17] Pandey VS Ouhelli H Elkhalfane A (1980) Observations on the epizootiology of Gasterophilus intestinalis and G. nasalis in horse in Morocco. Vet Parasitol. 7: 347– 356. 10.1016/0304-4017(92)90087-p 1502790

[18] Tavassoli M Bakht M (2012) *Gastrophilus* spp. myiasis in Iranian equine. Sci Parasitol. 13( 2): 83– 86.

[19] Liu SH Hu DF (2016b) Parasites observed in the proximal alimentary tract of a Przewalski's horse in China. Equine Vet Educ. 30: 20– 23.

[20] Liu SH Li K Hu DF (2016b) The incidence and species composition of *Gasterophilus* (Diptera, Gasterophilidae) causing equine myiasis in northern Xinjiang, China. Vet Parasitol. 217: 36– 38. 26827858 10.1016/j.vetpar.2015.12.028

[21] Zaheri BA Ronaghi H Youssefi MR Hoseini SM Omidzahir S Dozouri R Eshkevari SR Mousapour A (2015) *Gasterophilus pecorum* and *Habronema muscae* in Persian onager (*Equus* *hemionus onager*), histopathology and parasitology survey. Comp Clin Path. 24( 5): 1009– 1013.

